# Neuroleptic-induced acute respiratory distress syndrome

**DOI:** 10.1590/S1516-31802003000300007

**Published:** 2003-05-01

**Authors:** Francisco Garcia Soriano, Elcio dos Santos Oliveira Vianna, Irineu Tadeu Velasco

**Keywords:** Respiratory distress syndrome, Neuroleptic malignant syndrome, Adjuvants, anesthesia, Síndrome do desconforto respiratório, Síndrome maligna neuroléptica, Anestesia

## Abstract

**CONTEXT:**

A case of neuroleptic malignant syndrome and acute respiratory distress syndrome is presented and discussed with emphasis on the role of muscle relaxation, creatine kinase, and respiratory function tests.

**CASE REPORT:**

A 41-year-old man presented right otalgia and peripheral facial paralysis. A computed tomography scan of the skull showed a hyperdense area, 2 cm in diameter, in the pathway of the anterior intercommunicating cerebral artery. Preoperative examination revealed: pH 7.4, PaCO_2_ 40 torr, PaO_2_ 80 torr (room air), Hb 13.8 g/dl, blood urea nitrogen 3.2 mmol/l, and creatinine 90 μmol/l. The chest x-ray was normal. The patient had not eaten during the 12- hour period prior to anesthesia induction. Intravenous halothane, fentanyl 0.5 mg and droperidol 25 mg were used for anesthesia. After the first six hours, the PaO_2_ was 65 torr (normal PaCO_2_) with FiO_2_ 50% (PaO_2_/FiO_2_ 130), and remained at this level until the end of the operation 4 hours later, maintaining PaCO_2_ at 35 torr. A thrombosed aneurysm was detected and resected, and the ends of the artery were closed with clips. No vasospasm was present. This case illustrates that neuroleptic drugs can cause neuroleptic malignant syndrome associated with acute respiratory distress syndrome. Neuroleptic malignant syndrome is a disease that is difficult to diagnose. Acute respiratory distress syndrome is another manifestation of neuroleptic malignant syndrome that has not been recognized in previous reports: it may be produced by neuroleptic drugs independent of the manifestation of neuroleptic malignant syndrome. Some considerations regarding the cause and effect relationship between acute respiratory distress syndrome and neuroleptic drugs are discussed. Intensive care unit physicians should consider the possibility that patients receiving neuroleptic drugs could develop respiratory failure in the absence of other factors that might explain the syndrome.

## RESUMO

**CONTEXTO:**

Apresentamos um caso de síndrome neuroléptica maligna e de síndrome do desconforto respiratório do adulto relacionada ao uso do neuroléptico. Discutimos a possível relação clínica e as alterações de creatinino-quinase, relaxamento muscular e função respiratória.

**RELATO DE CASO:**

Paciente de 41 anos, homem, branco. Internado por otalgia e paralisia facial periférica à direita. A tomografia computadorizada de crânio mostrou uma área hiperdensa de 2 cm de diâmetro na região da artéria intercomunicante anterior. Os exames pré-operatórios revelaram: pH 7.4, PaCO_2_ 40 torr, PaO_2_ 80 torr (em ar ambiente), Hb 13.8 g/dl, nitrogênio uréico no plasma 3.2 mmol/l, e creatinina 90 mmol/l. A radiografia de tórax era normal. O paciente fez jejum de 12 horas previamente à cirurgia. Foram usados na anestesia: halotano, fentanil 0.5 mg e droperidol 25 mg (intravenosa). Após seis horas o paciente apresentava PaO_2_ 65 torr (PCO_2_ normal) sob FiO_2_ de 50% (relação PaO_2_/FiO_2_ 130) e permaneceu neste nível até o final da cirurgia. O achado intraoperatório foi de um aneurisma trombosado, que foi ressecado; o vaso foi clipado. Não havia vasoespasmo. Este caso ilustra que drogas neurolépticas podem causar síndrome maligna por neuroléptico e síndrome do desconforto respiratório do adulto em associação. Contudo, há possibilidade de que o neuroléptico ou o solvente da droga possam produzir síndrome do desconforto respiratório do adulto sem apresentar as manifestações de síndrome maligna por neuroléptico. Esta possibilidade é importante pois, nos pacientes com síndrome do desconforto respiratório do adulto que estejam recebendo neurolépticos, será necessário que se pense em sua substituição, especialmente se o paciente não apresentar outros fatores que possam explicar a síndrome do desconforto respiratório do adulto.

## INTRODUCTION

Neuroleptic malignant syndrome is characterized by muscle rigidity, hyperthermia, diaphoresis, fluctuating consciousness and rhabdomyolysis.^1-4^ Respiratory failure secondary to muscle rigidity, and aspiration and pulmonary embolism have been described.^5,6^ A case of acute respiratory distress syndrome associated with neuroleptic drug administration, with concomitant neuroleptic malignant syndrome, is reported.

## CASE REPORT

A 41-year-old man presented right otalgia and peripheral facial paralysis. A computed tomography scan of the skull showed a hyper-dense area, 2 cm in diameter, in the pathway of the anterior intercommunicating cerebral artery. Preoperative examination revealed: pH 7.4, PaCO_2_ 40 torr, PaO_2_ 80 torr (room air), hemoglobin (Hb) 13.8 g/dl, blood urea nitrogen 3.2 mmol/l, and creatinine 90 μmol/l. The chest x-ray was normal. The patient had not eaten during the 12-hour period prior to induction of anesthesia. Intravenous halothane, fentanyl 0.5 mg and droperidol 25 mg were used for anesthesia. After the first six hours, the PaO_2_ was 65 torr (normal PaCO_2_) with FiO _2_ 50% (PaO_2_/FiO_2_ 130), in volume-controlled mode with positive end-expiratory pressure (PEEP) 0 (zero) cmH_2_O. The PaO_2_ remained at this level until the end of the operation 4 hours later, and PaCO_2_ was kept near 35 torr during the surgical procedure. A thrombosed aneurysm was detected and resected, and the ends of the artery were closed with clips. No vasospasm was present.

After the operation, the patient was taken to the intensive care unit. Arterial pressure was 150/100 mmHg, heart rate was 110 beats/min and axillary temperature was 38.2° C. Psychomotor agitation, lead-pipe muscle rigidity of limbs and opistoton developed soon after coming out of the anesthesia. He was temporarily sedated by receiving 80 mg diazepam. It was necessary to progressively increase FiO_2_ to 100% with PaO_2_ 75 torr, PaCO_2_ 32 torr. The ventilation settings were changed to pressure-controlled ventilation, 7-8 ml/kg of tidal volume (V_T_), limiting the maximum pressure to 40 cmH_2_O and PEEP 14 cmH_2_O.

**Figure 1:**
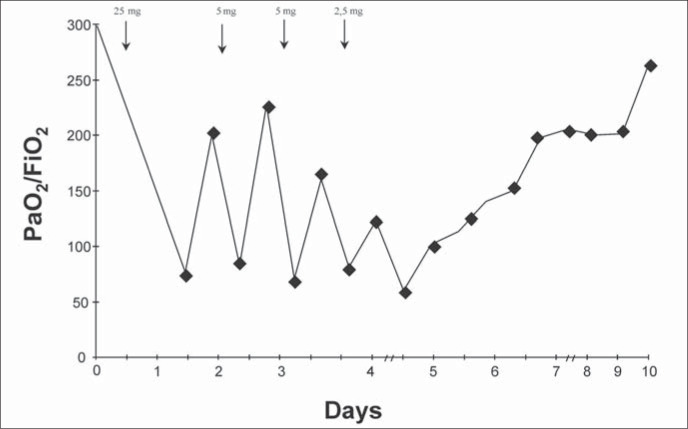
PaO_2_/FiO_2_ changes over the course of time. The arrows indicate neuroleptic administration after the surgical procedure.

Chest radiography demonstrated alveolar infiltration in the upper half of the right lung field. There had been no sign of aspiration since anesthesia, because he was intubated all the time. Alveolar recruitment maneuvers were realized with a discrete increase in PaO_2_ from 75 to 90 torr. Negative fluid balance was initiated. The PaO_2_/FiO_2_ ratio improved from 75 to 200 after 12 hours. Droperidol 5 mg was administered intravenously for the second time, due to persisting agitation and muscle rigidity. Six hours thereafter, the respiratory parameters worsened, confirmed by the drop in PaO_2_/FiO_2_ ratio to 83, in spite of keeping a negative fluid balance. The PaCO_2_ was maintained lower, at 35 torr throughout the treatment. The association between neuroleptic administration and lung worsening was repeated three more times (Figure 1).

On the third postoperative day, the axillary temperature rose to 39° C, but the limb extremities were cool and he had diaphoresis, a heart rate of 160 beats/min and arterial blood pressure of 170/120 mmHg. The temperature remained high for 18 hours in spite of the administration of fever-reducing agents and external cooling. Blood tests showed Na 136 mmol/l, K 4.2 mmol/l, creatine kinase 17,424 IU/l and 18,000 leukocytes/ml. A computed tomography scan of the skull failed to show cerebral hemorrhage, edema, or intracranial hypertension. The patient never presented convulsion.

Thus, the diagnosis of neuroleptic malignant syndrome was supported by the presence of hyperthermia, elevated creatine kinase, consciousness alterations, muscle rigidity and diaphoresis. The repetition of creatine kinase elevation and worsening of muscle rigidity concomitant to repeated neuroleptic administration was further strong support for this diagnosis. Blood, urinary, bronchoalveolar lavage and cerebrospinal fluid cultures were negative. Cytological analysis of cerebrospinal fluid showed erythrocytes 1200/ml, leukocytes 3/ml, protein 52 mg/dl and glucose 70 mg/dl.

Neuroleptic administration was then suspended, while sedation was maintained using benzodiazepines and fentanyl for 6 days and curare was necessary for the first 2 days. The neurological symptoms and temperature were monitored.

**Figure 2:**
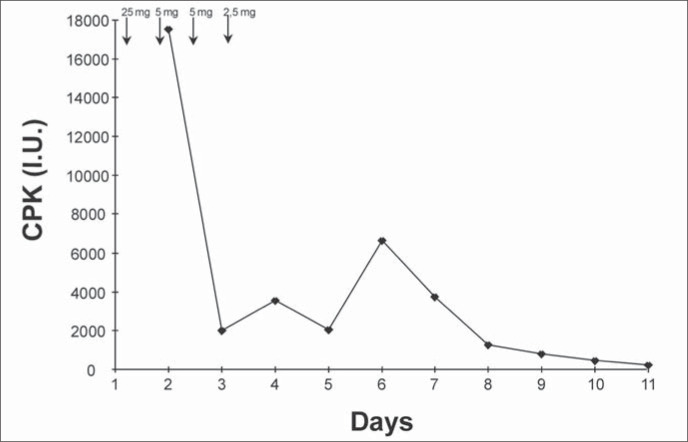
A notable elevation in creatine kinase (CPK) levels was related to neuroleptic drug administration (indicated by arrows).

On the fifth postoperative day, biochemical examination demonstrated creatine kinase 3571 IU/l, creatine kinase MB (CKMB) 167 IU/l, lactate dehydrogenase (LDH) 1054 IU/l, antimicrobial susceptibility testing (AST) 72 IU/l and alanine aminotransferase (ALT) 109 IU/l, and it was negative for myoglobinuria (Figure 2). The patient continued to suffer from respiratory failure. A hemodynamic study showed a cardiac index of 3.1 l/min.m^2^, central venous pressure of 11 mmHg, wedge pressure of the pulmonary artery 11 mmHg, systolic pulmonary artery 25 mmHg, diastolic pulmonary artery 15 mmHg, mean systemic arterial pressure 120 mmHg, systemic vascular resistance index 2,470 dynes/sec.cm^-5^.m^-2^, and pulmonary vascular resistance index of 234 dynes/sec.cm^-5^.m^-2^. On that day, a chest x-ray showed bilateral alveolar infiltration. The diagnosis of acute respiratory distress syndrome was established. No infection was ever found, and all bacteriological analyses were negative. The patient was maintained on positive end-expiratory pressure of 14 cmH_2_O, and maximum airway pressure was limited to 40 cmH_2_O with a minute volume of 18 l. Lung compliance was 20 ml/cmH_2_O. The neurological and pulmonary condition improved and he was extubated after 10 days.

On the 12^th^ postoperative day, biochemical examination showed creatine kinase of 40 IU/l (Figure 2), LDH 130 IU/l, AST 22 IU/l and ALT 21 IU/l. On the 14^th^ postoperative day he was transferred out of the intensive care unit, and on the 26^th^ postoperative day he was released from hospital. Two months thereafter, he had resumed his regular pattern of life.

## DISCUSSION

The neuroleptic malignant syndrome is an adverse effect from treatment with major tranquilizers. It is characterized by muscle rigidity and hyperthermia, and at least two of the following minor signs: diaphoresis, dysphagia, tremor, incontinence, altered mentation, mutism, tachycardia, elevated or labile blood pressure, leukocytosis and elevation of creatine phosphokinase.^1^ Often, the rectal temperature is over 41° C. Muscle rigidity and increased heat production result in acidosis and rhabdomyolysis. The creatine kinase level is elevated in 92% of cases and leukocytosis is common .^2^ Also, some authors have reported that the neuroleptic malignant syndrome developed within 8 hours of neuroleptic administration. Patients with organic brain disease are at high risk of developing the neuroleptic malignant syndrome and have a high rate of mortality (38.5%). Mortality is due to cardiovascular, pulmonary or renal collapse.^2,7^

Respiratory failure may be explained by several factors, such as aspiration, pulmonary embolus, pulmonary congestion due to heart failure, and hypoventilation due to muscle rigidity.^2-4^ However, this patient had acute respiratory distress syndrome without any of these possibilities. Pulmonary hypertension was ***not*** shown and there was no pressure gradient between pulmonary arterial wedge and pulmonary arterial diastolic pressures, which is commonly found in pulmonary embolus. Also, hemodynamic data showed ***normal*** pulmonary arterial wedge pressure, cardiac index, left ventricle systolic stroke work index and vascular systemic resistance, and such data discarded the possibility of heart failure or septic state. The PaCO_2_ was maintained at 35 mmHg, and thus there was ***no*** hypoventilation due to muscle rigidity. ***No*** aspiration episode was detected during the four episodes of respiratory worsening, since orotracheal tubing was maintained throughout this period. All fluids and samples analyzed for bacteria were negative. Therefore, there were no clinical criteria for the diagnosis of embolus, sepsis, heart failure or aspiration.

The repetitive worsening of PaO_2_/FiO_2_ related to the previous use of neuroleptic drugs over a period of 6-8 hours, as described in literature, and coupled with neuroleptic malignant syndrome, have led us to propose that the medication causes acute respiratory distress syndrome. Such a proposition is on a speculative basis. The speculation takes into account that whenever the PaO_2_/FiO_2_ ratio decreased, it was preceded by neuroleptic use over a period of 6-8 hours, and the patient presented a major clinical manifestation: the neuroleptic malignant syndrome.

Until the neuroleptic malignant syndrome was proposed, the patient was receiving repeated neuroleptic doses. This was producing repeated worsening of creatine kinase levels, muscle rigidity and the PaO_2_/FiO_2_ ratio. These facts corroborated the neuroleptic malignant syndrome diagnosis and the relationship between acute respiratory distress syndrome and neuroleptic malignant syndrome seen in this case. The chest x-ray with bilateral infiltration; PaO_2_/FiO_2_ ratio < 150; positive end-expiratory pressure and lung compliance producing a Murray score^8^ of 3.25; and the hemodynamic study (wedge pressure 11 mmHg) all corroborated the diagnosis of acute respiratory distress syndrome .^9,7^ Furthermore, the clinical evolution did not involve congestion, sepsis or aspiration. The suspension of neuroleptic drugs with the use of benzodiazepine and curare led to significant improvement with respect to muscle relaxation, normalization of temperature and subsequent normalization of biochemical markers and blood leukocyte count.^10^

This case illustrates that neuroleptic drugs ***may*** cause association between neuroleptic malignant syndrome and acute respiratory distress syndrome. Neuroleptic malignant syndrome is not easily diagnosed, which make it difficult to establish its relationship with other manifestations, such as acute respiratory distress syndrome, for example. We can raise two possible associations between acute respiratory distress syndrome and the neuroleptic malignant syndrome. Firstly, acute respiratory distress syndrome may be produced independently of the presence of neuroleptic malignant syndrome. Secondly, acute respiratory distress syndrome could be another neuroleptic malignant syndrome manifestation. Our main possibilities is to bring up a discussion regarding this possible association, so as to generate some ideas that researchers in this field could try to reproduce in experimental models, to verify what is really taking place.

On the other hand, it is common for physicians to treat patients with adverse reactions to drugs. In cases similar to ours, if there is a doubt about the drug, it can be stopped and other without such an effect can be substituted. Therefore, the intensive care unit physician should consider the possibility of discontinuing neuroleptic use among patients who are receiving neuroleptic drugs and develop respiratory failure.
